# KBN2202 Suppresses Gonadal White Adipose Tissue Expansion in Female Mice Fed a High-Fat Diet

**DOI:** 10.3390/ijms27020627

**Published:** 2026-01-08

**Authors:** Moonhang Kim, Jeong-Hyeon Heo, Seok Hwan Chang, Sun-Young Lee, Jihun Kim, Moon-Geun Shin, Jong Sung Kim, Mi Ran Choi, Sang-Rae Lee

**Affiliations:** 1 Efficacy Test Center for Mental & Behavioral Disorders, Ajou University Hospital, Suwon 16499, Republic of Korea; mook1052@ajou.ac.kr (M.K.); tjrghksekrzj@ajou.ac.kr (S.H.C.); sun02lee@ajou.ac.kr (S.-Y.L.); mgshin@ajou.ac.kr (M.-G.S.); kjs0829@ajou.ac.kr (J.S.K.); 2 Department of Pharmacology, Ajou University School of Medicine, Suwon 16499, Republic of Korea; hjh2g@ajou.ac.kr (J.-H.H.); soulmate415@ajou.ac.kr (J.K.); 3 BK21 R&E Initiative for Advanced Precision Medicine, Suwon 16499, Republic of Korea; 4 Department of Biomedical Sciences, Graduate School of Ajou University, Suwon 16499, Republic of Korea; 5 Laboratory Animal Research Center, Ajou University School of Medicine, Suwon 16499, Republic of Korea

**Keywords:** white adipose tissue, obesity, high-fat diet, GLP-1, TNF-α

## Abstract

Obesity treatments increasingly target multiple pathways beyond appetite suppression. We evaluated KBN2202, a salicylate-derived small molecule, in a high-fat diet (60% kcal from fat) mouse model using female and male C57BL/6J mice treated for 8 weeks with oral KBN2202 (20 mg/kg/day) or a matched-volume vehicle (1% DMSO/PBS). Body weight was recorded weekly, and food intake was measured daily; serum hormones and cytokines, adipose tissue histology, and open-field behavior were assessed at the end of the study. Under our experimental conditions, HFD increased body weight and gonadal white adipose tissue (gWAT)/brown adipose tissue (BAT) mass in females, whereas males showed only modest HFD-associated weight gain and did not develop a clear obesity phenotype. KBN2202 significantly reduced peri-ovarian gWAT mass and adipocyte size without altering overall body weight. In females, circulating glucagon-like peptide-1 (GLP-1) increased, uncoupling protein 1 (UCP1) in gWAT showed a non-significant upward trend, and serum TNF-α was selectively decreased, while MCP-1 and IL-1β were unchanged. Locomotor activity was unaltered, and anxiety-like behavior was reduced. Male mice did not show comparable adipose effects. These findings indicate depot-specific, peripheral modulation of adipose remodeling, hormonal balance, and inflammatory tone by KBN2202, supporting its further investigation as an adipose-targeted metabolic modulator complementary to incretin-based therapies.

## 1. Introduction

Obesity is defined as an abnormal or excessive accumulation of body fat and is regarded as a complex disease that leads to various metabolic and functional impairments beyond simple weight gain. The development of obesity is primarily driven by high-calorie diets, physical inactivity, imbalanced energy metabolism, and genetic factors [1]. In particular, high-fat and high-sugar diets cause a chronic energy surplus, which promotes adipocyte hyperplasia and hypertrophy, resulting in increased fat mass and inflammatory changes [2]. Visceral fat, such as gonadal white adipose tissue (gWAT), plays a central role in obesity-related metabolic dysfunction by secreting pro-inflammatory cytokines and contributing to insulin resistance [3].

Excessive fat accumulation contributes to a wide range of health problems. Direct consequences include joint pain, sleep apnea, and reduced physical mobility due to increased body weight [4]. Indirectly, obesity is strongly associated with a higher risk of chronic conditions such as type 2 diabetes, hypertension, dyslipidemia, cardiovascular disease, non-alcoholic fatty liver disease, and certain types of cancer [5,6]. Notably, the pathological expansion of adipocytes promotes immune cell infiltration and the secretion of pro-inflammatory cytokines such as TNF-α, IL-1β, MCP-1, and IL-6, leading to a state of low-grade chronic inflammation [7,8]. This inflammatory condition is recognized as a central mechanism that exacerbates the development and progression of obesity-related metabolic disorders [2,7,9].

In recent years, obesity treatment has evolved beyond simple weight loss to focus on metabolic improvement and long-term safety [10]. For example, semaglutide, a glucagon-like peptide-1 receptor agonist (GLP-1 RA), has demonstrated significant weight reduction by suppressing appetite and stimulating insulin secretion [11,12,13]. However, these agents still present several limitations. Reported issues include gastrointestinal side effects (e.g., nausea, vomiting, diarrhea, constipation), loss of muscle mass, poor tolerability, and weight regain after treatment discontinuation [11,14,15,16]. These challenges are largely attributed to their single-pathway mechanism of action and their limited capacity to modulate broader metabolic processes. Therefore, there is an increasing demand for multi-targeted therapeutic strategies that regulate both adipose tissue-specific pathways and systemic metabolic hormones more comprehensively.

Salicylic acid, a prototypical nonsteroidal anti-inflammatory drug (NSAID), has long been used to treat inflammatory conditions [17]. It is known to inhibit the NF-κB and IKKβ pathways [18,19], thereby suppressing the production of pro-inflammatory cytokines [20,21]. Beyond its anti-inflammatory actions, emerging evidence suggests that salicylic acid also exerts beneficial metabolic effects in obesity models, including improvements in hepatic steatosis, insulin resistance, and body weight regulation [21,22]. These mechanistic insights have led to increasing interest in salicylate-based derivatives as potential therapeutic agents for modulating obesity-related pathophysiology [23,24].

Based on these considerations, we hypothesized that KBN2202, a novel small-molecule compound structurally derived from salicylic acid, may serve as a multi-targeted therapeutic candidate capable of modulating adipose tissue expansion and metabolic hormone pathways. This hypothesis is supported by the dual role of salicylates in both anti-inflammatory signaling and energy balance regulation. In this context, we specifically focused on evaluating whether KBN2202 could overcome key limitations of current anti-obesity drugs by exerting broader adipose tissue- and hormone-centered mechanisms, rather than relying solely on appetite suppression or body weight reduction.

## 2. Results

### 2.1. Effects of KBN2202 on Body Weight and Dietary Intake

**Figure 1:**
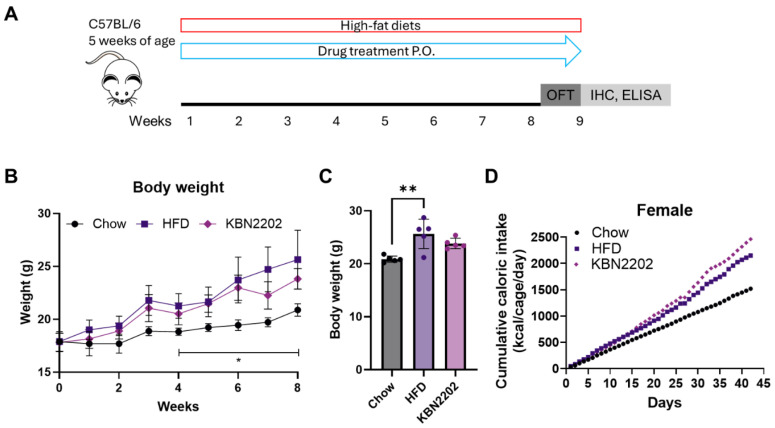
Effects of KBN2202 on body weight and dietary intake in HFD-fed female mice (male data are presented in Supplementary Figure S1): (**A**) Schematic overview of the 8-week experimental design involving HFD feeding and daily oral administration of KBN2202 (20 mg/kg). (**B**) Weekly body weight measurements over the 8-week study period. HFD-fed mice showed a significant increase in body weight compared with the chow group, whereas KBN2202-treated females showed a modest attenuation of weight gain on average, but this difference did not reach statistical significance. The bracket and asterisks indicate statistical significance for the chow vs. HFD comparison at the indicated time point. (**C**) Final body weight at week 8 was analyzed using the Kruskal–Wallis test. (**D**) Cumulative energy intake (kcal) during the 6-week monitoring period (weeks 3–8), calculated from daily food disappearance per cage (g/day) × diet energy density (kcal/g). Each trace represents one cage (5 mice/cage), and each point represents one day. Because intake was measured at the cage level with one cage per group and may be biased by feed shredding/spillage, intake data are presented descriptively without inferential statistics. Data are presented as mean ± SD (*n* = 5 per group and 1 cage/group for panel (**D**)). Statistical significance was determined by two-way RM-ANOVA followed by Tukey’s post hoc test (**B**) and analyzed using the Kruskal–Wallis test (**C**) followed by Dunn’s multiple-comparison test (* *p* < 0.05, ** *p* < 0.01).

Eight weeks of high-fat diet (HFD) feeding increased body weight relative to chow-fed controls, with a robust effect in females (Figure 1B,C). In males, HFD-induced weight gain was modest and did not produce a clear obesity phenotype (Supplementary Figure S1A,B). In female mice, KBN2202 administration was associated with a modest attenuation of weight gain on average, but this difference did not reach statistical significance (Figure 1B,C). In male mice, KBN2202 did not attenuate HFD-associated body-weight gain relative to the HFD vehicle group; terminal body weight in the KBN2202 group exceeded that of chow controls (Supplementary Figure S1A,B). Cage-level cumulative energy intake appeared broadly similar between the HFD and KBN2202 groups in females and males (Figure 1D; Supplementary Figure S1C), with no apparent treatment-associated difference in overall energy intake.

### 2.2. Suppression of gWAT Expansion and Adipocyte Hypertrophy by KBN2202

**Figure 2:**
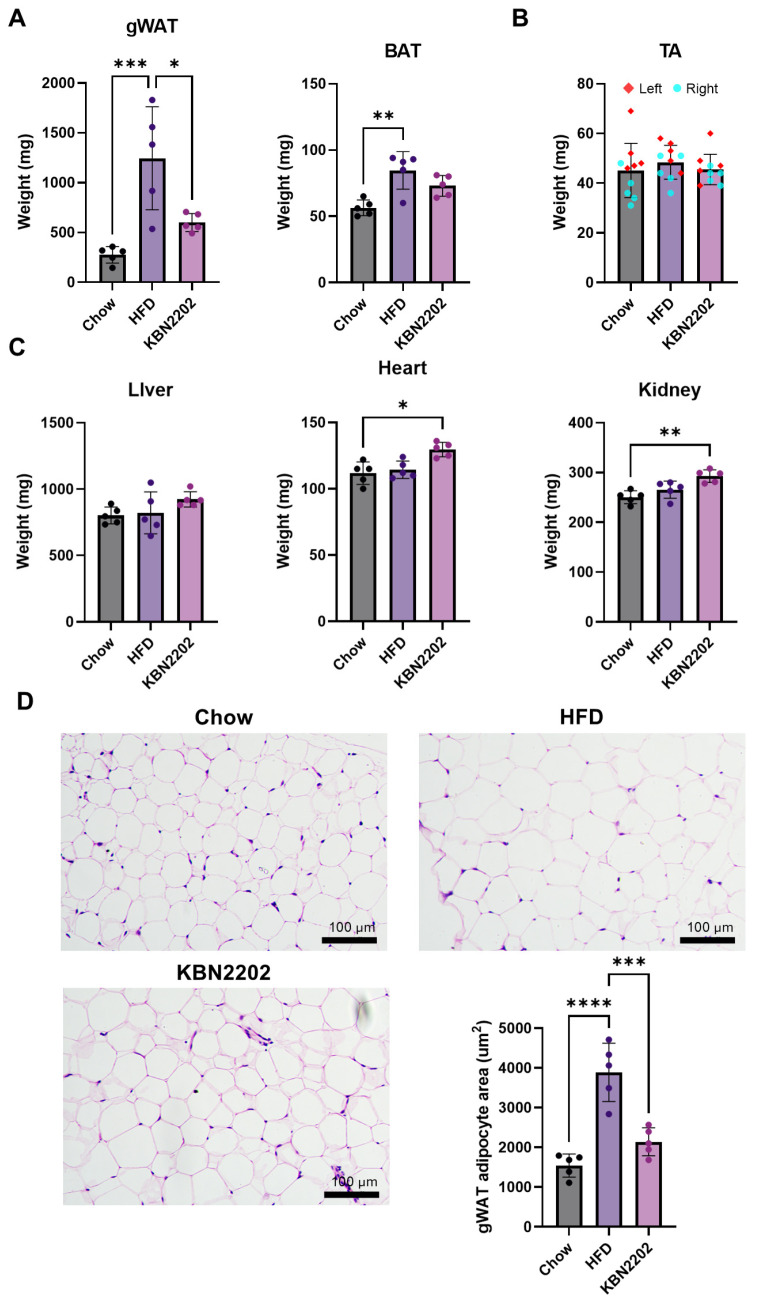
Effects of KBN2202 on adipose tissue, organ weight, and adipocyte hypertrophy in females: (**A**) Absolute weights of gWAT and BAT. KBN2202 significantly reduced gWAT weight compared with the HFD group, whereas BAT weight was unchanged. (**B**) Absolute weight of TA muscle (left and right), showing no significant differences between groups. (**C**) Absolute weights of liver, heart, and kidneys were comparable among groups. (**D**) Representative H&E-stained images of gWAT and quantification of adipocyte area. The HFD group showed a marked increase in adipocyte size, which was significantly reduced by KBN2202 treatment. Scale bar = 100 μm. Data shown are from female mice; corresponding male data are provided in Supplementary Figure S1. Data are presented as mean ± SD (*n* = 5 per group). Statistical significance was assessed using one-way ANOVA followed by Tukey’s post hoc test, except for gWAT- and BAT-related endpoints, which were analyzed using the Kruskal–Wallis test followed by Dunn’s multiple-comparison test (* *p* < 0.05, ** *p* < 0.01, *** *p* < 0.001, **** *p* < 0.0001).

To evaluate the effects of KBN2202 on diet-induced obesity, the weights of adipose tissues and major organs were measured. In female mice, HFD feeding significantly increased the weights of gWAT and brown adipose tissue (BAT), indicating enhanced adipogenesis. KBN2202 treatment significantly suppressed gWAT weight gain by approximately 48% compared with the HFD group, whereas BAT weight remained unchanged (Figure 2A). To assess possible changes in muscle mass, the tibialis anterior (TA) muscle was analyzed, and no significant differences in TA weight were observed among groups (Figure 2B). Although KBN2202 markedly reduced gWAT weight, total body weight remained unchanged, likely because modest increases in other organs (e.g., the liver, heart, and kidneys) offset the reduction in gWAT (Figure 1B,C and Figure 2A–C). In contrast, male mice did not exhibit a comparable response to KBN2202 (Supplementary Figure S1D–F). Accordingly, subsequent adipose analyses focused on female gWAT (Figure 2A), and adipocyte size in gWAT was quantified. After 8 weeks of HFD feeding, the average adipocyte area increased approximately threefold (from ~1000 μm^2^ to ~3800 μm^2^), indicating marked adipocyte hypertrophy (Figure 2D). KBN2202 treatment significantly reduced adipocyte size by nearly 50% compared with the HFD group.

### 2.3. Modulation of Thermogenic Markers and GLP-1 Levels by KBN2202

**Figure 3:**
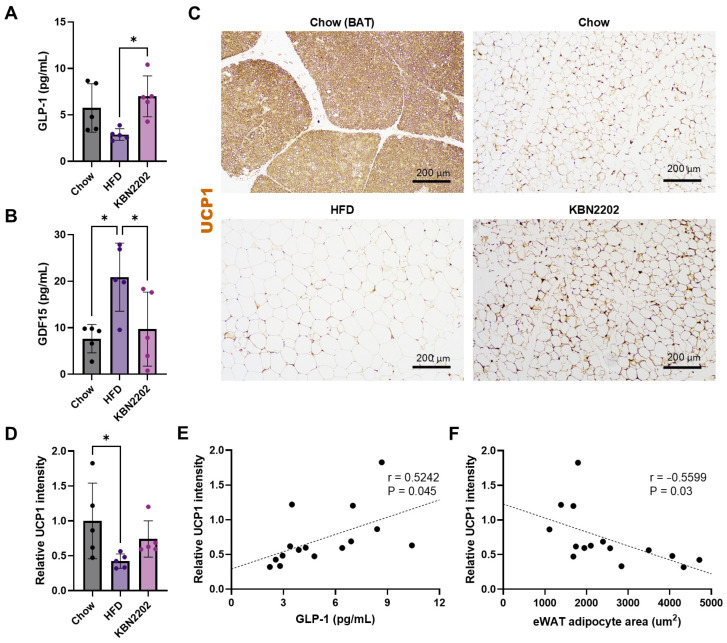
Effects of KBN2202 on serum GLP-1 and GDF15 levels and gWAT UCP1 expression in female mice, and their correlations: (**A**) Serum GLP-1 levels were significantly elevated in the KBN2202-treated group compared with the HFD group (*p* < 0.05). (**B**) Serum GDF15 levels were markedly elevated in the HFD group and were reduced in the KBN2202-treated group. (**C**) Representative immunohistochemical staining of UCP1 in gWAT. A normal BAT section stained for UCP1 was included as a positive control (labeled ‘Chow (BAT)’ in the representative images). Scale bar = 200 μm. (**D**) Quantification of UCP1 staining intensity in gWAT. KBN2202 increased UCP1 immunoreactivity by ~1.5-fold versus HFD, but this trend did not reach significance (*p* = 0.07). (**E**) Correlation between UCP1 intensity and serum GLP-1 levels (*n* = 15, r = 0.5242, *p* = 0.045). (**F**) Correlation between adipocyte area and UCP1 intensity (*n* = 15, r = −0.5599, *p* = 0.03). (Associations; not causal inference.) Data are shown as mean ± SD (*n* = 5 per group). Statistical significance was determined by one-way ANOVA followed by Tukey’s post hoc test (**A**,**B**) and Kruskal–Wallis test (**D**) followed by Dunn’s multiple-comparison test. (* *p* < 0.05). Correlation analyses (**E**,**F**) were performed using Pearson’s correlation with pooled data from all groups (total *n* = 15).

To evaluate the effects of KBN2202 on thermogenic and hormonal pathways, serum GLP-1 and growth differentiation factor 15 (GDF15) levels, as well as uncoupling protein 1 (UCP1) expression in gWAT, were examined in female mice. Serum GLP-1 levels were significantly higher in the KBN2202 group than in the HFD group (*p* < 0.05) (Figure 3A). In contrast, serum GDF15 levels, which were markedly elevated in the HFD group, were significantly reduced following KBN2202 treatment, returning to values comparable to those of the chow group (*p* < 0.05) (Figure 3B). Immunohistochemical analysis showed that UCP1 expression was markedly decreased in the HFD group compared with the chow group (Figure 3C). KBN2202 tended to increase UCP1 immunoreactivity in gWAT compared with the HFD group, but this difference did not reach statistical significance (*p* = 0.07; Figure 3D). Pearson correlation analysis revealed a positive correlation between UCP1 expression in gWAT and serum GLP-1 levels (Figure 3E), indicating an association between these variables. Conversely, a significant negative correlation was observed between adipocyte area and UCP1 expression (Figure 3F), consistent with an inverse association between UCP1 immunoreactivity and adipocyte size.

### 2.4. Effects of KBN2202 on Systemic Inflammation in HFD-Fed Female Mice

**Figure 4:**
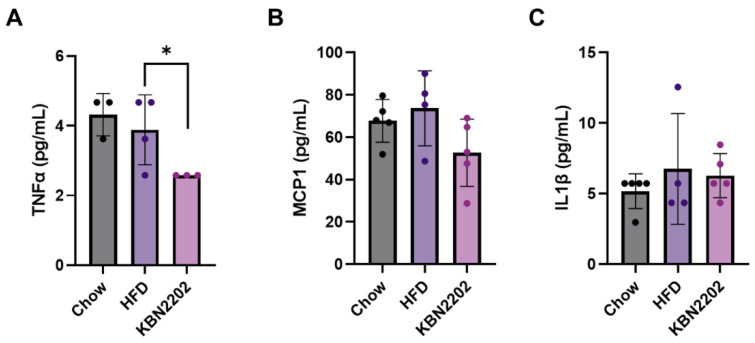
Effects of KBN2202 on pro-inflammatory cytokines in female mice. Serum concentrations of (**A**) TNF-α, (**B**) MCP-1, and (**C**) IL-1β were quantified using a multiplex immunoassay. TNF-α levels were significantly reduced in the KBN2202 group compared with the HFD group (*p* < 0.05). MCP-1 and IL-1β levels did not differ significantly among groups. Data are presented as mean ± SD of quantifiable values (≥limit of blank (LOB)). Values below the assay’s LOB were classified as non-detectable (ND) (indistinguishable from blank/background) and are reported as ND. The number of ND samples is as follows: TNF-α, chow *n* = 2/5, HFD *n* = 1/5, KBN2202 *n* = 2/5; MCP-1, chow *n* = 0/5, HFD *n* = 1/5, KBN2202 *n* = 0/5; IL-1β, chow *n* = 0/5, HFD *n* = 1/5, KBN2202 *n* = 0/5. ND values were not assigned a numerical concentration (i.e., not treated as 0 pg/mL) and were not included in the calculation of mean ± SD or in inferential testing. Statistical significance was evaluated using an unpaired two-tailed Student’s *t*-test comparing the HFD and KBN2202 groups (chow group shown for reference). * *p* < 0.05.

To assess whether KBN2202 influences systemic inflammatory profiles in an HFD-induced model, serum concentrations of eight cytokines (MCP-1, IFN-γ, IL-1β, IL-4, IL-6, IL-10, IL-13, and TNF-α) were measured using a multiplex bead-based assay in female mice (Figure 4). Most cytokines were below the limit of blank (LOB) across all groups, indicating minimal systemic inflammatory activation under the current experimental conditions. Among detectable analytes, TNF-α showed a consistent reduction following KBN2202 treatment (*p* < 0.05; Figure 4A), whereas MCP-1 and IL-1β remained unchanged (Figure 4B,C). In the KBN2202 group, two samples were below the LOB, and the remaining three produced identical plotted concentrations because median fluorescence intensity (MFI) signals on the Luminex^®^ 100/200™ platform are displayed in 0.5-unit increments. The underlying fluorescence values, however, differed slightly (CV < 3%), confirming that these were independent biological replicates.

### 2.5. Effects of KBN2202 on Locomotor Activity and Anxiety-like Behavior in the Open-Field Test

**Figure 5:**
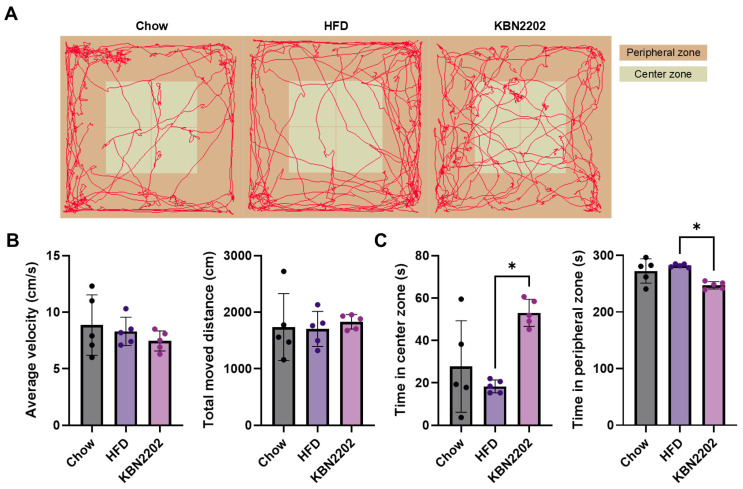
Effects of KBN2202 on locomotor activity and anxiety-like behavior in the open-field test: (**A**) Representative locomotor tracks during the final 5 min of the 10 min test period. (**B**) Mean locomotor speed (cm/s) and total distance traveled (cm). No significant differences were observed among groups in overall locomotor activity. (**C**) Time spent in the center and peripheral zones. KBN2202-treated mice spent significantly more time in the center zone and less time in the peripheral zone compared with the HFD group (*p* < 0.05). Data are presented as mean ± SD (*n* = 5 per group). Statistical significance was assessed using one-way ANOVA followed by Tukey’s post hoc test (* *p* < 0.05).

To evaluate the effects of KBN2202 on locomotion and anxiety-related behavior, mice were subjected to the open-field test (OFT) after eight weeks of HFD feeding. Representative locomotor tracks demonstrated no apparent differences in general movement patterns among groups (Figure 5A). Quantitative analysis confirmed that neither mean locomotor speed nor total distance traveled differed significantly across groups (Figure 5B), indicating no impact on baseline locomotor activity. However, KBN2202-treated mice spent significantly more time in the center zone and less time in the peripheral zone compared with the HFD group (*p* < 0.05; Figure 5C).

## 3. Discussion

Recent advances in obesity therapeutics have shifted toward multi-targeted approaches that address the limitations of current pharmacological agents, moving beyond weight loss strategies. For instance, although GLP-1 RAs are effective in reducing body weight, their efficacy appears to be reduced in patients with type 2 diabetes compared with non-diabetic individuals [25]. To overcome these limitations, combination therapies based on entero-pancreatic hormones are currently under development [26]. Additionally, to mitigate the adverse effect of muscle mass loss associated with GLP-1 RAs, preclinical studies are investigating the co-administration of GLP-1 RAs with bimagrumab, an antibody that blocks activin type II receptors [27]. These examples reflect a growing interest in combinatorial strategies that modulate multiple metabolic pathways simultaneously. Therefore, we investigated the effects of KBN2202, a novel small molecule structurally derived from salicylic acid with reported anti-inflammatory [17,18,19] and metabolic regulatory properties [21,24,28], in an HFD-feeding paradigm.

In the present study, KBN2202 reduced HFD-induced adiposity endpoints in female mice, whereas the male cohort did not show comparable adipose responses. Notably, under our housing conditions, male mice exhibited only modest HFD-associated body-weight gain and did not develop a clear obesity phenotype (Supplementary Figure S1A–F), which limits the interpretability of treatment effects in males. Susceptibility to diet-induced obesity in C57BL/6J mice has been reported to vary with sex and with the age at which high-calorie feeding is initiated [29], and males may show relatively greater resistance when obesogenic feeding begins around puberty. However, we did not measure individual-level energy intake or energy expenditure in the present study; therefore, the basis for the weaker male HFD response cannot be determined from this dataset. Although body-weight trajectories and general cage conditions were monitored throughout the study and no conspicuous abnormalities (e.g., aggressive fighting or marked body-weight loss in individual animals) were observed, future studies incorporating individual metabolic phenotyping (e.g., individual food intake, locomotor activity, and indirect calorimetry-based energy expenditure) will be required to more rigorously quantify HFD responsiveness in male mice and to enable a more definitive assessment of KBN2202 efficacy in this sex.

HFD consumption induces body-weight gain through a chronic positive energy balance, leading to excessive caloric intake, increased adipogenesis, and reduced energy expenditure [30,31]. Mechanistically, this process promotes adipocyte hypertrophy and hyperplasia, particularly in visceral fat depots such as gWAT, which contribute to systemic inflammation and insulin resistance [32]. HFD can also evoke adaptive thermogenic responses; however, thermogenic capacity in BAT and beige adipocytes is often impaired, limiting their ability to counteract obesity [33,34]. Current anti-obesity agents such as GLP-1 RA primarily reduce body weight by suppressing appetite through central mechanisms and secondarily improve glycemic control [11,35]. While appetite suppression plays a critical role in energy balance, direct regulation of adipose tissue expansion and function is also essential in combating obesity.

In this context, KBN2202 significantly suppressed HFD-induced gWAT expansion and adipocyte hypertrophy in female mice. Open-field testing did not reveal differences in overall locomotor activity, arguing against a major contribution of altered baseline activity to body-weight dynamics. Cage-level cumulative energy intake during the monitoring window appeared broadly comparable between the HFD and KBN2202 groups; however, because intake was assessed at the cage level (one cage per group) and can be influenced by food handling and recovery bias, these data are descriptive and cannot exclude subtle individual-level differences in energy balance. KBN2202 increased circulating GLP-1 levels, while UCP1 expression in gWAT showed a non-significant upward trend. Importantly, these findings do not constitute direct evidence of enhanced thermogenesis, as energy expenditure and other functional thermogenic readouts were not assessed. Accordingly, the observed correlations among GLP-1, UCP1, and adipocyte size should be interpreted as associative rather than mechanistic.

GDF15 is a stress-responsive cytokine belonging to the transforming growth factor-β (TGF-β) superfamily [36]. It is induced by various cellular stressors, including inflammation, hypoxia, mitochondrial dysfunction, and nutrient excess, and has been implicated in the regulation of energy balance and body weight [36,37]. Elevated circulating GDF15 levels are often observed in pathological states such as cancer, cardiovascular disease, and metabolic disorders, where it acts as an anorexigenic signal via its receptor GFRAL in the hindbrain to suppress appetite [36]. Based on these properties, recombinant GDF15 and GDF15 analogs have been explored as potential therapeutic agents for obesity, demonstrating robust weight loss effects in preclinical and early-phase clinical studies [38,39]. Several studies have reported that HFD feeding increases circulating GDF15 in mice, supporting its use as a metabolic stress marker in diet-induced obesity models [40,41]. In our study, serum GDF15 levels were increased in HFD-fed mice, and KBN2202 reduced elevated GDF15 levels toward those observed in chow groups. This reduction may reflect attenuation of stress-associated signaling; however, the functional implications for energy balance and the mechanistic link to KBN2202 cannot be determined from the present dataset. Importantly, the elevation of GDF15 under HFD despite lower food intake by mass may reflect diet-induced metabolic stress and/or compensatory anorexigenic signaling. In addition, because HFD is more energy-dense than chow and energy expenditure was not assessed, lower gram intake does not necessarily indicate reduced caloric intake or altered energy balance.

Pro-inflammatory cytokines such as TNF-α, IL-1β, and MCP-1 are key mediators of adipose tissue remodeling, promoting lipogenesis, hypertrophy, and insulin resistance [42]. However, in the present study, serum levels of these cytokines were not significantly elevated in HFD-fed mice despite marked adipocyte hypertrophy and gWAT expansion. This pattern may reflect the relatively short duration of HFD exposure (8 weeks) or an early metabolic stage in which adipose remodeling precedes overt systemic cytokine elevation [43,44,45]. Notably, KBN2202 selectively reduced serum TNF-α concentrations. While salicylate derivatives can influence inflammatory signaling pathways such as IKKβ/NF-κB [46], the relevant signaling events and local inflammatory changes within adipose tissue were not directly assessed here. Therefore, the TNF-α finding supports selective modulation of an inflammatory mediator but does not, by itself, establish broad systemic anti-inflammatory efficacy.

To exclude the possibility that KBN2202 influenced body-weight dynamics through changes in general locomotor performance, we performed the open-field test, which provides indices of basal activity and exploratory locomotion. KBN2202 treatment did not alter total distance traveled or mean locomotor speed, indicating no detectable effect on overall locomotor activity. Increased time spent in the center zone was observed, suggesting reduced anxiety-like behavior. However, because central readouts were not assessed, we did not interpret these behavioral changes as evidence for a CNS-mediated appetite-regulating mechanism in the present study.

In conclusion, KBN2202 suppressed HFD-induced gWAT expansion and adipocyte hypertrophy in female mice and was associated with increased circulating GLP-1, reduced GDF15, and selective reduction in TNF-α, while UCP1 in gWAT showed only a non-significant upward trend. Male mice did not develop a robust HFD-induced obesity phenotype under our housing conditions and did not show comparable adipose responses to KBN2202, limiting interpretation in males. Because energy expenditure/indirect calorimetry, glucose tolerance or insulin sensitivity, and mechanistic pathway readouts were not assessed, our conclusions are limited to adipose remodeling and the serum endpoints measured in this study (GLP-1, GDF15, and cytokines, including TNF-α). Future studies incorporating comprehensive metabolic phenotyping, direct thermogenic assessments, and conditions that reliably induce comparable obesity phenotypes across sexes will be required to define systemic and mechanistic effects of KBN2202.

## 4. Materials and Methods

### 4.1. Animal Model of High-Fat Diet-Induced Obesity

Four-week-old male and female C57BL/6J mice were purchased from DBL Co., Ltd. (Daejeon, Republic of Korea) and housed under controlled conditions (21 ± 2 °C, 12-h light/dark cycle) with ad libitum access to food and water. After a one-week acclimation period, five-week-old male and female mice were randomly assigned to three groups (5 mice per cage; 1 cage per group for each sex): normal chow (Chow), HFD, and HFD plus KBN2202 (KBN2202). All procedures were identical across sexes; sex-stratified analyses were prespecified. Female data are presented in the main figures; male data are provided in Supplementary Figure S1. The normal chow diet was Teklad Irradiated Global 18% Protein Rodent Diet (2918; Teklad Diets, Inotiv, West Lafayette, IN, USA; energy density 3.1 kcal/g). The HFD provided 60% of total calories from fat (D12492; Saeron Bio, Cheonan-si, Republic of Korea; energy density 5.24 kcal/g). Both male and female groups were maintained on their respective diets for eight weeks to induce obesity. The animal study was approved by the Institutional Animal Care and Use Committee of the Advanced Medical Bio Research Center (IACUC-2024-009) and conducted in accordance with institutional and national guidelines.

### 4.2. Drug Treatment

KBN2202 is a salicylic acid derivative (molecular formula C19H19NO4; molecular weight 325.35 g/mol) synthesized and provided by KAISER Bio Ltd. (Gyeongsan, Republic of Korea) and formulated as previously described [47]. Briefly, KBN2202 was initially dissolved in 1 mL of DMSO (200 mg/mL) and subsequently diluted with 99 mL of PBS to yield a final solution containing 1% DMSO and 2 mg/mL of the compound. This solution was administered orally at a dose of 20 mg/kg/day via oral gavage using a 20-gauge feeding needle. Mice in the Chow and HFD groups received daily oral gavage of vehicle (1% DMSO in PBS) at a matched volume. Drug administration began concurrently with the high-fat diet and continued for eight weeks (Figure 1A). This concurrent initiation was intended to evaluate the preventive efficacy of KBN2202 against diet-induced adipose expansion and inflammation, rather than to assess a therapeutic effect after obesity establishment.

### 4.3. Physiological Measurements and Tissue Collection

Body weight was recorded weekly throughout the experimental period. Food intake was assessed as daily food disappearance at the cage level (experimental unit = cage) for 6 weeks starting 2 weeks after diet/drug initiation (weeks 3–8). A pre-weighed amount of diet was supplied every 24 h, and the remaining food was collected and weighed the next day. Pellets dispersed into bedding were retrieved using a fine sieve and included whenever possible; however, complete recovery of shredded/spilled pellets was not always possible. To account for differences in energy density between diets, daily food disappearance (g/cage/day) was converted to energy intake (kcal/cage/day) using manufacturer-provided energy density (kcal/g), and cumulative energy intake was calculated per cage. After the final drug administration (week 8), behavioral assessment was performed using the OFT to evaluate locomotor activity and anxiety-like behavior. Two days after behavioral testing, mice were fasted for 6 h and subsequently anesthetized with Avertin (2,2,2-tribromoethanol, 250 mg/kg, intraperitoneally; T1420, Tokyo Chemical Industry Co., Ltd., Tokyo, Japan), followed by euthanasia via exsanguination. Blood samples were collected in Microtainer^®^ SST tubes (365967; BD, Franklin Lakes, NJ, USA). Major organs—including the liver, heart, kidneys, gWAT, BAT, and TA muscle—were excised and weighed for further analysis. Serum was isolated by centrifugation at 3000 rpm for 15 min at 25 °C and stored at −80 °C until further use.

### 4.4. Quantification of Serum Cytokines by ELISA and Multiplex Analysis

Serum levels of GLP-1 (BMS2194; Invitrogen, Waltham, MA, USA) and GDF15 (MGD150; R&D Systems, Minneapolis, MN, USA) were measured using a commercial ELISA kit according to the manufacturer’s instructions. All samples were analyzed in duplicate, and optical density was measured using a microplate reader (INNO-S, LTek, Sungnam, Republic of Korea). Serum cytokines, including MCP-1, IFN-γ, IL-1β, IL-4, IL-6, IL-10, IL-13, and TNF-α, were quantified using a multiplex bead-based assay (Mouse XL Cytokine Premixed Kit; R&D Systems, FCSTM20) on a Luminex^®^ 100/200™ platform. Samples were diluted 1:2 in assay buffer and analyzed in duplicate according to the manufacturer’s instructions. MFI values were used for back-calculation of concentrations according to a 5-parameter logistic standard curve. On this platform, MFI signals are displayed in 0.5-unit increments, which can lead to discretized concentration outputs appearing as identical values when the true fluorescence intensities are very close. Each sample was analyzed in duplicate, and values below the assay’s limit of blank (LOB) were considered non-detectable (ND) and were reported as ND rather than as quantitative concentration values. The number of ND samples per group is provided for transparency.

### 4.5. Histology and Immunohistochemistry

Tissue samples were fixed in 4% paraformaldehyde (PFA) for 24 h at 4 °C, embedded in paraffin, and sectioned at a thickness of 4 µm. Sections were deparaffinized, rehydrated, and stained using standard protocols for hematoxylin and eosin (H&E) staining. Adipocyte size was quantified from H&E-stained gWAT sections using ImageJ software version 1.54 (NIH, Bethesda, MD, USA). For each animal, three non-overlapping fields (200× magnification) were randomly selected from the central region of the tissue section. Cell boundaries were manually delineated using the “Freehand selection” tool, and adipocyte area (µm^2^) was measured after automated thresholding with consistent parameters across all samples. Approximately 150–200 adipocytes were analyzed per animal. Image analysis was performed by two independent observers blinded to group allocation. For immunohistochemical analysis of thermogenic activity, sections were deparaffinized, rehydrated, and subjected to antigen retrieval by heating in 10 mM sodium citrate buffer (pH 6.0) at 95 °C for 20 min, followed by cooling at room temperature. Endogenous peroxidase activity was quenched with 2% hydrogen peroxide for 10 min, and non-specific binding was blocked with 5% normal goat serum for 1 h at room temperature. Sections were then incubated overnight at 4 °C with a rabbit anti-UCP1 primary antibody (1:500, ab10983; Abcam, Cambridge, UK). Signal detection was performed using the ImmPRESS^®^-HRP Horse Anti-Rabbit IgG Polymer Reagent (MP-7401; Vector Laboratories, Newark, CA, USA) and a DAB substrate kit (SK-4103; Vector Laboratories, Newark, CA, USA). Interscapular brown adipose tissue (BAT) was used as a positive control for UCP1, and negative controls were prepared by omitting the primary antibody. Representative images were acquired using an OLYMPUS CKX3-HOUN bright-field microscope (Olympus Corporation, Tokyo, Japan). UCP1 expression levels were quantified by measuring mean DAB signal intensity in gWAT sections using ImageJ with consistent thresholding across samples.

### 4.6. Open-Field Test (OFT)

Locomotor activity and anxiety-like behavior were assessed using the open-field test in a dedicated sound-attenuated behavioral testing room. To minimize stress caused by external noise, continuous white noise (60 ± 5 dB) was played throughout the experiment. The open-field apparatus consisted of a square arena (40 × 40 × 40 cm) made of opaque white acrylic, placed under uniform illumination of approximately 150 lux provided by an overhead LED light source. Prior to testing, each mouse was acclimated to the behavioral testing room for 30 min to minimize novelty-induced stress. Mice were then individually placed in the center of the arena and allowed to freely explore for 10 min. A ceiling-mounted camera (Basler acA1300, Ahrensburg, Germany) positioned 100 cm above the arena was connected to an automated video-tracking system (EthoVision XT, version 11.5; Noldus Information Technology, Wageningen, The Netherlands) for continuous tracking of the animal’s center of mass at a sampling rate of 15 frames per second. The center zone was defined as a 20 × 20 cm square in the middle of the arena. Between trials, the arena was thoroughly wiped with odorless wet wipes to remove residual scent cues without introducing stress-inducing odors. Behavioral parameters analyzed included total distance traveled (cm), mean velocity (cm/s), and time spent in the center and peripheral zones (s). To avoid early exploratory bias, behavioral data were analyzed from the final 5 min of the 10 min session. All data were analyzed offline by an investigator blinded to treatment allocation using the same tracking software.

### 4.7. Statistical Analysis

Data are presented as mean ± SD unless otherwise indicated. Statistical analyses were performed using GraphPad Prism 10 (GraphPad Software, San Diego, CA, USA). Normality and homogeneity of variances were assessed using the Shapiro–Wilk and the Brown–Forsythe test, respectively. For single-time-point outcomes, one-way ANOVA followed by Tukey’s multiple-comparison test was used when assumptions were met; when normality and/or variance-homogeneity assumptions were violated, the Kruskal–Wallis test followed by Dunn’s multiple-comparison test was applied. For serum cytokines, the planned pairwise comparison between the HFD and KBN2202 groups was evaluated using an unpaired two-tailed Student’s *t*-test. Weekly body weight was analyzed using a two-way repeated-measure ANOVA with group (Chow, HFD, KBN2202) and time as factors, including the group × time interaction, followed by Tukey’s multiple-comparison test comparing groups at each time point. Correlation analyses were performed using Pearson’s correlation. Food/energy intake was assessed at the cage level and is presented descriptively without inferential statistics because the cage was the experimental unit. Statistical significance was set at *p* < 0.05. Analyses were performed separately for females and males.

## Data Availability

The datasets generated and analyzed in this study are available from the corresponding author upon reasonable request.

## Supplementary Materials

The following supporting information can be downloaded at: https://www.mdpi.com/article/10.3390/ijms27020627/s1.

## Abbreviations

The following abbreviations are used in this manuscript:

BAT	Brown adipose tissue
gWAT	Gonadal white adipose tissue
GDF15	Growth differentiation factor 15
GLP-1	Glucagon-like peptide-1
GLP-1 RA	GLP-1 receptor agonist
HFD	High-fat diet
LOB	Limit of blank
MFI	Median fluorescence intensity
NSAID	Nonsteroidal anti-inflammatory drug
OFT	Open-field test
TA	Tibialis anterior
UCP1	Uncoupling protein 1
