# Supplementary material for: KBN2202 Suppresses Gonadal White Adipose Tissue Expansion in Female Mice Fed a High-Fat Diet
Source: Int J Mol Sci. 2026 Jan 8;27(2):627. doi: 10.3390/ijms27020627 (PMC12841312; doi:10.3390/ijms27020627)
Supplement: Supplementary file 1 [file ijms-27-00627-s001.zip › ijms-4029111-supplementary.pdf]

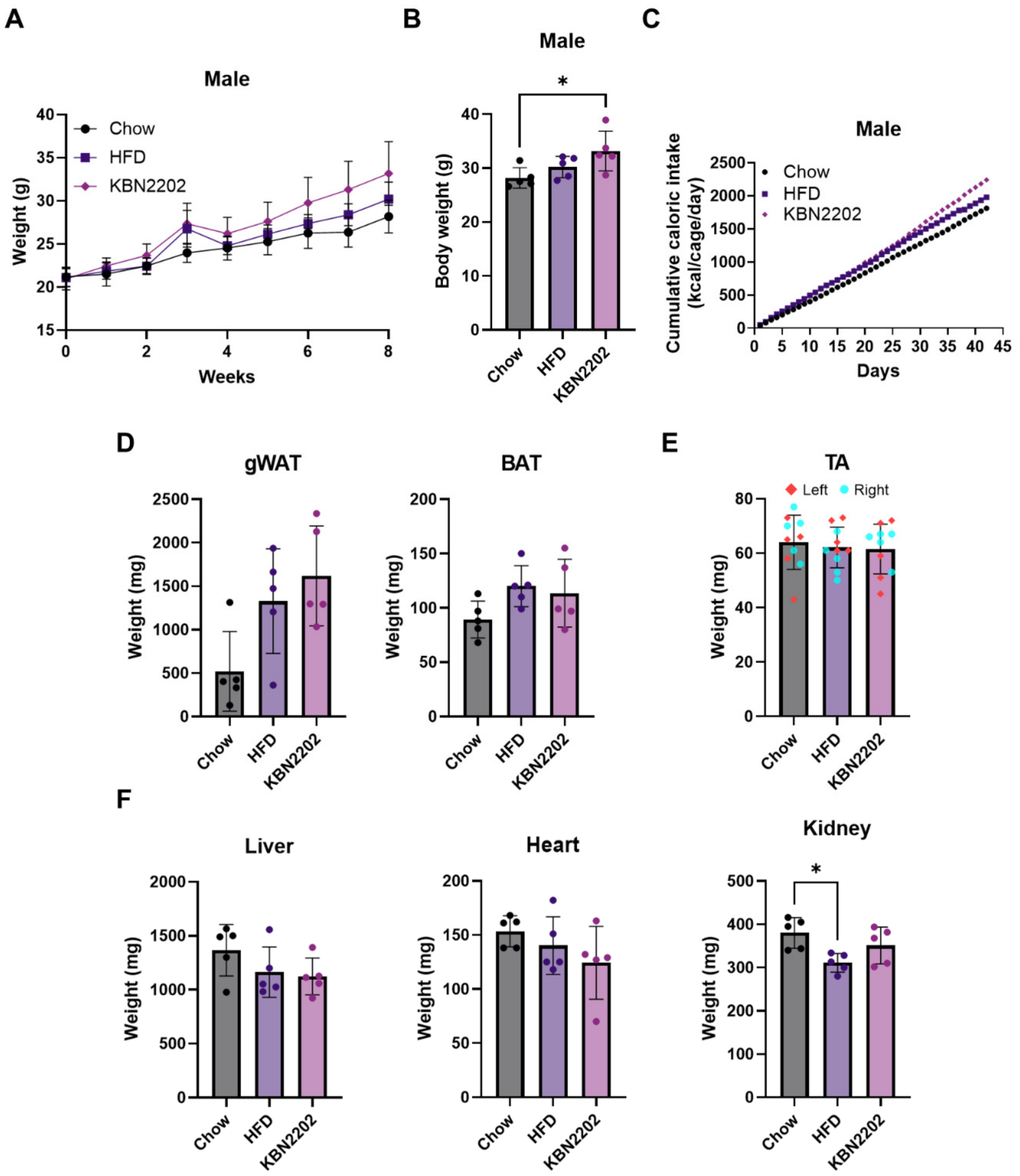

Figure S1. Effects of KBN2202 on body weight, food intake, and tissue weights in HFD-fed m ale mice. (A) Weekly body weight changes during the 8-week study period in male mice. (B) Final body weight at the end of the 8-week treatment period. (C) Cumulative caloric intake, c alculated as the sum of daily food consumption over the study period. (D) Weights of gonada l white adipose tissue (gWAT) and brown adipose tissue (BAT). (E) Tibialis anterior (TA) mus cle weight, shown separately for left and right muscles. (F) Weights of liver, heart, and kidne ys at the end of the study. Data are presented as mean ± SD (n = 5 per group). Statistical sig nificance was determined by one-way ANOVA followed by Tukey's post hoc test (*p < 0.05, * ***p < 0.0001).

Supplementary Table S1. Summary of statistical tests and p-values for all experimental outcomes (by sex)

End point	Sex	Groups compared	N	*P* value		Passed	Test used	Multiple comparisons
				Shapiro-Wilk	Brown-Forsythe			
Body weight (weekly)	F	Chow	5			Yes	2-way RM-ANOVA	Tukey
		HFD	5			Yes		
		KBN2202	5			Yes		
Final body weight	F	Chow	5	0.7773	0.0169	No	Kruskal-Wallis	Dunn's
		HFD	5	0.5039		Yes		
		KBN2202	5	0.4770		Yes		
gWAT weight	F	Chow	5	0.4660	0.0309	No	Kruskal-Wallis	Dunn's
		HFD	5	0.8286		Yes		
		KBN2202	5	0.4901		Yes		
BAT weight	F	Chow	5	0.8267	0.7341	Yes	Kruskal-Wallis	Dunn's
		HFD	5	0.0280		No		
		KBN2202	5	0.3229		Yes		
TA weight	F	Chow	5	0.3235	0.3528	Yes	One-way ANOVA	Tukey
		HFD	5	0.8435		Yes		
		KBN2202	5	0.0808		Yes		
Liver weight	F	Chow	5	0.7654	0.1858	Yes	One-way ANOVA	Tukey
		HFD	5	0.7815		Yes		
		KBN2202	5	0.0717		Yes		
Heart weight	F	Chow	5	0.7754	0.8393	Yes	One-way ANOVA	Tukey
		HFD	5	0.5443		Yes		
		KBN2202	5	0.6483		Yes		
Kidney weight	F	Chow	5	0.8537	0.9254	Yes	One-way ANOVA	Tukey
		HFD	5	0.2191		Yes		
		KBN2202	5	0.4131		Yes		
Adipocyte area weight	F	Chow	5	0.2773	0.2461	Yes	One-way ANOVA	Tukey
		HFD	5	0.8662		Yes		
		KBN2202	5	0.9233		Yes		
GLP-1	**F**	Chow	5	0.1006	0.2054	Yes	One-way ANOVA	Tukey
		HFD	5	0.5657		Yes		
		KBN2202	5	0.5204		Yes		
GDF15	F	Chow	5	0.0832	0.2771	Yes	One-way ANOVA	Tukey
		HFD	5	0.3955		Yes		
		KBN2202	5	0.3311		Yes		
UCP1	F	Chow	5	0.5976	0.1533	Yes	Kruskal-Wallis	Dunn's
		HFD	5	0.6175		Yes		
		KBN2202	5	0.0051		No		
TNF-α	F	Chow	3		0.1573	No	Student's t-test	
		HFD	4	0.2730		Yes		
		KBN2202	3	Invalid input data				
MCP-1	F	Chow	5	0.6666	0.6619	Yes	One-way ANOVA	Tukey
		HFD	4	0.478		Yes		
		KBN2202	5	0.6967		Yes		
IL-1β	F	Chow	5	0.0001	0.4263	No	Kruskal-Wallis	Dunn's
		HFD	4	0.0338		No		

		KBN2202	5	0.8140		Yes		
Average velocity	F	Chow	5	0.5252	0.1705	Yes	One-way ANOVA	Tukey
		HFD	5	0.4584		Yes		
		KBN2202	5	0.8981		Yes		
Total moved distance	F	Chow	5	0.2997	0.8714	Yes	One-way ANOVA	Tukey
		HFD	5	0.8993		Yes		
		KBN2202	5	0.1811		Yes		
Time in center zone	F	Chow	5	0.7071	0.1249	Yes	One-way ANOVA	Tukey
		HFD	5	0.3490		Yes		
		KBN2202	5	0.7183		Yes		
Time in peripheral zone	F	Chow	5	0.7091	0.1238	Yes	One-way ANOVA	Tukey
		HFD	5	0.3846		Yes		
		KBN2202	5	0.7277		Yes		
Body weight (weekly)	M	Chow	5			Yes	2-way RM-ANOVA	Tukey
		HFD	5			Yes		
		KBN2202	5			Yes		
Final body weight	M	Chow	5	0.1508	0.6517	Yes	One-way ANOVA	Tukey
		HFD	5	0.2928		Yes		
		KBN2202	5	0.6209		Yes		
gWAT weight	M	Chow	5	0.0531	0.7919	Yes	One-way ANOVA	Tukey
		HFD	5	0.5429		Yes		
		KBN2202	5	0.2220		Yes		
BAT weight	M	Chow	5	0.9956	0.5266	Yes	One-way ANOVA	Tukey
		HFD	5	0.5727		Yes		
		KBN2202	5	0.4840		Yes		
TA weight	M	Chow	5	0.6628	0.7449	Yes	One-way ANOVA	Tukey
		HFD	5	0.7007		Yes		
		KBN2202	5	0.2729		Yes		
Liver weight	M	Chow	5	0.1757	0.9011	Yes	One-way ANOVA	Tukey
		HFD	5	0.1080		Yes		
		KBN2202	5	0.5596		Yes		
Heart weight	M	Chow	5	0.0945	0.7941	Yes	One-way ANOVA	Tukey
		HFD	5	0.1800		Yes		
		KBN2202	5	0.2904		Yes		
Kidney weight	M	Chow	5	0.1764	0.5273	Yes	One-way ANOVA	Tukey
		HFD	5	0.7750		Yes		
		KBN2202	5	0.2236		Yes		
